# Investigation on YSZ- and SiO_2_-Doped Mn-Fe Oxide Granules Based on Drop Technique for Thermochemical Energy Storage

**DOI:** 10.3390/molecules29091946

**Published:** 2024-04-24

**Authors:** Yan Ma, Kai Wang, Sikai Liang, Zhongqing Li, Zhiyuan Wang, Jun Shen

**Affiliations:** School of Energy and Power Engineering, University of Shanghai for Science and Technology, Shanghai 200093, China; 18395588645@163.com (Y.M.); wang213410@163.com (K.W.); liang18339426475@163.com (S.L.); llq10162457@163.com (Z.L.); kennyshen@vip.163.com (J.S.)

**Keywords:** thermochemical energy storage, Mn-Fe oxide granules, doping, packed-bed reactor, sintering inhibition

## Abstract

The Mn-Fe oxide material possesses the advantages of abundant availability, low cost, and non-toxicity as an energy storage material, particularly addressing the limitation of sluggish reoxidation kinetics observed in pure manganese oxide. However, scaling up the thermal energy storage (TCES) system poses challenges to the stability of the reactivities and mechanical strength of materials over long-term cycles, necessitating their resolution. In this study, Mn-Fe granules were fabricated with a diameter of approximately 2 mm using the feasible and scalable drop technique, and the effects of Y_2_O_3_-stabilized ZrO_2_ (YSZ) and SiO_2_ doping, at various doping ratios ranging from 1–20 wt%, were investigated on both the anti-sintering behavior and mechanical strength. In a thermal gravimetric analyzer, the redox reaction tests showed that both the dopants led to an enhancement in the reoxidation rates when the doping ratios were in an appropriate range, while they also brought about a decrease in the reduction rate and energy storage density. In a packed-bed reactor, the results of five consecutive redox tests showed a similar pattern to that in a thermal gravimetric analyzer. Additionally, the doping led to the stable reduction/oxidation reaction rates during the cyclic tests. In the subsequent 120 cyclic tests, the Si-doped granules exhibited volume expansion with a decreased crushing strength, whereas the YSZ-doped granules experienced drastic shrinkage with an increase in the crushing strength. The 1 wt% Si and 2 wt% Si presented the best synthetic performance, which resulted from the milder sintering effects during the long-term cyclic tests.

## 1. Introduction

Thermal energy storage (TES) is one of the promising solutions for addressing the intermittency and fluctuation of renewable energy sources, such as solar energy, wind energy, tidal energy, et al. [1], which can be classified into three categories based on the mechanisms, i.e., sensible heat storage (SHS) [2], latent heat storage (LHS) [3], and thermochemical heat/energy storage (TCES) [4]. Thermochemical energy storage (TCES) systems based on metal oxides have emerged as prominent solutions due to their advantages of a high energy storage density and the utilization of ambient air both as the gaseous reagent and heat transfer fluid (HTF), compared with other conventional energy storage systems [5]. Among those metal oxide candidates, for example, Co_3_O_4_/CoO [6], CuO/Cu_2_O [7], BaO_2_/BaO [8], and Mn_2_O_3_/Mn_3_O_4_ [9], manganese oxides possess the advantages of cost-effectiveness, abundance, non-toxicity, and environmental benignity. In particular, the Mn_2_O_3_/Mn_3_O_4_ system is a kind of relatively more researched TCES system when compared with the Co_3_O_4_/CoO system with the highest energy storage density, which is always controversial due to its potential carcinogenic properties [10]. The primary limitations of Mn_2_O_3_/Mn_3_O_4_ systems are the sluggish reoxidation rate and gradual degradation resulting from sintering during multiple cycles, as well as the thermal hysteresis characteristics of the redox reactions, the onset temperature gap of which is about approximately 200 °C. All this leads to a decline in the cyclability and exergy efficiency [11,12,13]. The chemical modification strategy, especially doping Fe cations, can effectively enhance the re-oxidation kinetics and lower the hysteresis when compared with the morphology modification [14,15]. The doping of the Fe cation has been extensively demonstrated in numerous studies as a highly effective approach to expedite the rate of the oxidation reaction and enhance the cycle stability [11,13,16,17,18,19,20]. Examples of Mn/Fe oxides with varying molar ratios, arranged in descending order of the energy storage density, include 3:1 (271 J/g) [19], 2:1 (233 J/g) [11], 4:1 (219 J/g) [17], and 1:2 (~200 J/g) [21].

The TCES system, which is based on solid–gas reactions, has utilized various reactor types including fluidized-bed reactors, moving-bed reactors, and fixed-bed reactors [22,23,24]. The fixed-bed reactors stand out among these reactor configurations due to the cost-effectiveness, the scalability, and the ability of effectively mitigating particle attrition and erosion when compared to the fluidized-bed reactors. However, the main drawbacks of a fixed-bed reactor involve the pressure drop caused by large quantities of finely powdered material and the sintering effect that occurs during a prolonged exposure to high temperatures [15]. These factors would result in gas channeling and a decrease in the reaction rate in the practical operation. Additionally, the inherent poor heat transfer within the fixed-bed can be addressed by employing air as both a reactant and a heat transfer fluid (HTF). Therefore, the selection of the appropriate thermal storage materials in the fixed-bed reactor is crucial and all factors such as metal oxide compositions, the shape, and the size should be synthetically considered. Lots of investigations have been conducted to enhance the energy storage density of oxide materials by pelletization or granulation [20,21], extruded modules [25,26], and coated structures [27,28]. In the case of a fixed-bed reactor, utilizing mm-sized spherical particles offers advantages such as a reduced void fraction and enhanced homogeneity.

During operation at a high temperature, the micro- and macro-structure of the material may be deteriorated with the cycled reductions and oxidations due to the multitude of stresses (chemical, mechanical, and thermal) experienced in a reactor. Wokon et al. demonstrated that the morphology of Mn-Fe oxide particles, prepared by a build-up granulation technique without any support or binder, underwent significant changes due to severe sintering issues in the 100 high-temperature cyclic tests. Consequently, this resulted in a decrease in the rate of the oxidation reaction and cycle stability. Additionally, a substantial increase in the particle volume was observed after the redox cycles, resulting in the decreased density and heightened fragility of the particles [19]. Xiang et al. observed a 15% decrease in the energy storage capacity of a honeycomb module weighing 110 g, composed of Mn/Fe oxides with a molar ratio of 4:1, after 100 redox cycles [26].

The resolution of these issues has been pursued through various endeavors, including the optimization of particle preparation methods and the addition of support materials as the sintering inhibitor. The addition of 10 wt% alumina was found to significantly alleviate the expansion of the Co_3_O_4_ honeycomb upon the redox cyclic process, thereby maintaining the superior thermo-mechanical stability of the macro-structural compared to that of the pure Co_3_O_4_ honeycomb [25]. Preisner et al. studied the impact of incorporating 20 wt% of various supporting materials, namely ZrO_2_, CeO_2_, and TiO_2_, on the mechanical strength of manganese–iron oxide particles prepared by the build-up granulation technique in a moving-bed reactor [29]. Compared to the undoped particles, which exhibited a high tendency for agglomeration and fracture, the incorporation of ZrO_2_ and CeO_2_ can effectively alleviate this phenomenon and enhance the attrition strength, while TiO_2_ doping is not suitable as the formation of a stable phase occurs. Gan et al. investigated the impact of the SiC doping of (Mn_0_._8_Fe_0_._2_)_2_O_3_ particles, prepared by the extrusion–spheronization technique, on the attrition resistance and long-term reaction cycle performance of the oxides [23]. They reported that incorporating 0.5–5 wt% SiC into Mn-Fe particles enhanced the attrition resistance and long-term cyclability of the Mn-Fe particles, although the reaction performance was slightly reduced. Gigantino et al. investigated the anti-sintering effect of yttria-stabilized zirconia (YSZ) on the porous CuO-based granules, which were fabricated by the drop technique and tested in a packed reactor for two separate sets of 30 cycles under isobaric and isothermal conditions [30]. The results demonstrated that the doped granules presented the significant mitigation of agglomeration when the doping ratio exceeded 50 wt%. Bielsa et al. further identified the critical parameters of the granule synthesis process based on the previous drop technique, as modified by Gigantino et al., and investigated the TCES performance of Si-doped manganese oxide particles in a lab-scale packed-bed reactor [31]. The author claimed that increasing the temperature to 1100 °C can enhance the mechanical strength by 30%. In addition, Si-doped manganese oxide particles exhibited complete re-oxidation behavior during 100 cyclic tests, indicating that Si helps mitigate the sintering effects, and the results were consistent with their prior findings [32].

In summary, the spherical manganese–iron oxide particles exhibit significant potential as a promising candidate for high-temperature thermal energy storage (TCES) systems in fixed-bed reactors. This also imposes corresponding requirements on the strength and reactivity of the particles under the high-temperature conditions, which can be enhanced through appropriate preparation and doping strategies. In this work, we fabricated Mn-Fe oxide granules with a molar ratio of 3:1 via the drop technique which is a novel, feasible, and scalable granule synthesis method. The effect of doping different mass ratios of SiO_2_ (at 1 wt%, 2 wt%, 5 wt%, and 10 wt%) and YSZ (at 5 wt%, 10 wt%, and 20 wt%) on the characteristics of Mn-Fe granules was investigated in this study. Firstly, we examined the redox performance of two kinds of dopants with varying doping mass ratios using a STA and a lab-scale packed-bed reactor, respectively. Secondly, the doped granules were subjected to 120 redox cyclic tests and were sampled every 30 cycles to assess the TECS performance evolution in a simultaneous thermal analyzer (STA). Corresponding analytical techniques were employed before and after the cycles to identify micro- and macro-structural changes. The results obtained from this experiment on granules modification have, to some extent, contributed to the advancement of upscaling TCES systems, which is also applicable to other metal oxide pairs.

## 2. Results and Discussion

### 2.1. Effect of Dopants on Redox Reaction Characteristics

**Figure 1:**
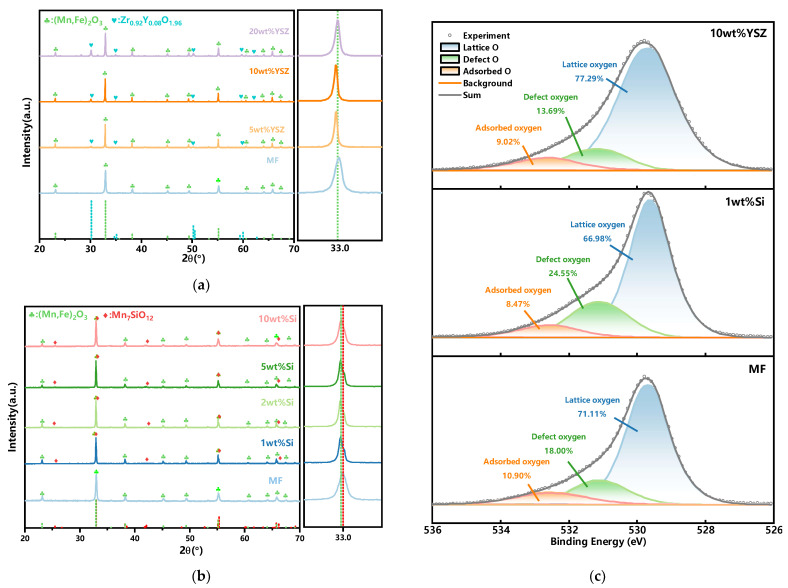
Analyses of the phase composition and chemical state of O of the doped granules. (**a**) YSZ-doped granules (left) and a magnification of the region of 32–34° (right) (green dotted line: (Mn,Fe)_2_O_3_); (**b**) Si-doped granules (left) and a magnification of the region of 32–34° (right) (green dotted line: (Mn,Fe)_2_O_3_), red dotted line: Mn_7_SiO_12_). (**c**) O 1s XPS spectra of MF, 1 wt% Si, and 10 wt% YSZ.

The characterization of the phase compositions and the chemical states of the oxygen of the doped Mn-Fe granules are shown in Figure 1. As depicted in Figure 1a, only two distinct phases, (Mn, Fe)_2_O_3_ (ICDD; PDF-2; #41-1442) and Zr_0_._92_Y_0_._08_O_1_._96_ (ICDD; PDF-2; #48-0224), are present in the synthesized granules, indicating that no new phase is formed during the calcination process. Furthermore, the intensity of the YSZ diffraction peak gradually increases with the doping ratio. Although some shifts of the characteristic peak of (Mn, Fe)_2_O_3_ are observed with the YSZ doping, the peak shifts are irregular. For example, the peak gradually shifts to the low diffraction angle region with the increase in the doping ratio from 0 to 10 wt%, whereas it shifts to the high diffraction angle region with a further increase in the ratio to 20 wt%. In the XRD tests, many factors could result in the peak shift, such as the structure strain from the doping of a heterogeneous atom, the inhomogeneity and impurity in the sample, the morphological characteristics of the sample (powder, coating, and film), the conditions of the test instrument, etc. Generally, the peak shift would show a regular trend with heterogeneous atoms doping as the change in the cell volume because of the replacement of the host atom by the doped atom [33,34,35]. Therefore, the shifts of the characteristic peak in Figure 1a may not only be ascribed to the doping effects. In order to clarify the reason for irregular shifts when YSZ is doped in the XRD pattern, a lot of work is thus needed. As the TCES performance is the main concern in this study, a detailed analysis of the XRD peak shift has been out of the scope of this work. In the next work, a more precise characterization should be conducted to investigate the doping effect of YSZ on the characteristic peak of (Mn, Fe)_2_O_3_. In the case of Si-doped granules, a new complex phase, braunite Mn_7_SiO_12_ (ICDD; PDF-2; #41-1367), is observed with increasing the SiO_2_ content, which also exhibits a characteristic peak next to Mn_2_O_3_ in the XRD patterns, as depicted in Figure 1b. The magnification of the region of 32–34° shows the characteristic peak overlap of (Mn, Fe)_2_O_3_ and Mn_7_SiO_12_. Previous studies have reported that the presence of this braunite phase is undesirable because of its exceptional stability within the investigated temperature range [32,36]. 

The O species in MF, 1 wt% Si, and 10 wt% YSZ is further analyzed using XPS, and the corresponding O 1s spectra are depicted in Figure 1c. Three distinct peaks are observed in the deconvoluted O 1s spectra. The predominant peak at ~530 eV is consistent with lattice oxygen. The peak at ~531 eV can be attributed to defect oxygen or surface oxygen ions, and a peak at ~533 eV is indicative of adsorbed oxygen species such as hydroxyl (OH^−^) and carbonate (CO_3_^2−^). Furthermore, the content of lattice oxygen, defect oxygen, and adsorbed oxygen is calculated based on the area of those oxygen species in the XPS spectra. The lattice oxygen content in MF is 71.11%, while it is increased to 77.29% for 10 wt% YSZ due to the high oxygen ion conductivity of YSZ. The incorporation of YSZ has been demonstrated to enhance the concentration of lattice oxygen and facilitate the conversion of a portion of adsorbed oxygen into surface lattice oxygen, which serves as the rate-determining step in the redox reaction [37]. The concentration of defect oxygen is increased to 24.55% when Si is doping. This means that an approximate amount of Si may lead to the enhanced adsorption of oxygen at the surface as more defect oxygen species would facilitate the adoption of oxygen in the gas phase. Therefore, the reoxidation process could be improved with the more facilitated transport of oxygen ions within the crystal lattice [38,39]. 

**Figure 2:**
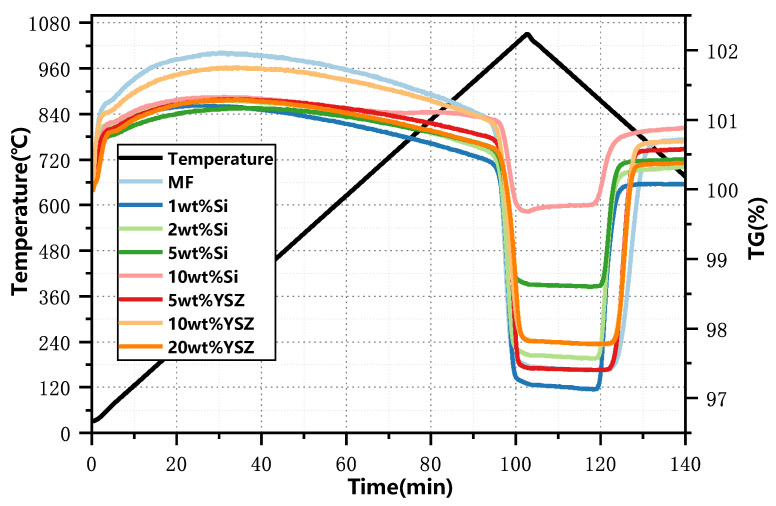
TG profiles of the doped granules at a heating rate of 10 °C min^−1^.

**Figure 3:**
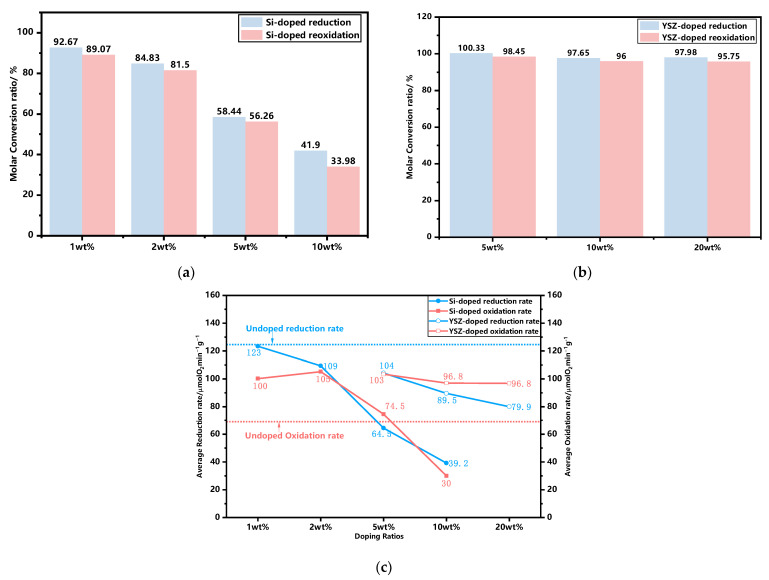
The influence of doping on the redox reaction rate. (**a**) Molar conversion ratio of Si-doped granules; (**b**) molar conversion ratio of YSZ-doped granules; (**c**) the average reduction and oxidation rate at different doping ratios.

The thermogravimetric (TG) curves of synthesized granules, incorporating various dopants and ratios, were experimentally obtained within a temperature range of 650 °C to 1050 °C using a ramp rate of 10 °C min^−1^, as illustrated in Figure 2. The results obtained from the TG curves are summarized in Table 1. It is found that the discrepancy in the mass change between the experimental and theoretical results for YSZ-doped samples is negligible, with a maximum deviation of only 0.07%, indicating that YSZ serves an inert function in redox reactions. However, the observed mass changes of Si-doped granules exhibit a significant deviation from their corresponding theoretical values, which gradually diminishes with an increase in the Si content. The influence of doping on the redox reaction rate is depicted in Figure 3. It is found that when the doping ratio of Si is 10 wt%, the reduction conversion is only 41.9% and the reoxidation conversion is 33.98%, as depicted in Figure 3a. The reason for this significant reduction in the reaction conversions can be attributed to the formation of a stable manganese silicon phase known as braunite Mn_7_SiO_12_, which consumes a portion of the active oxide. It is also supported by the XRD analyses in Figure 1b. 

As depicted in Figure 3c, the average reduction and oxidation rates of YSZ exhibit a gradual decline as the doping ratio increases from 5 wt% to 20 wt%, resulting in a minimum rate of 79.86 μmol O_2_ min^−1^ g^−1^ for reduction, while stabilizing at 96.78 μmol O_2_ min^−1^ g^−1^ for oxidation. The overall trend for Si doping exhibits similarities to that of YSZ doping, while within the doping range of 5–10 wt%, Si doping demonstrates a higher susceptibility to adverse effects in terms of both reduction and oxidation rates compared to YSZ doping at the same scale. The average reduction and oxidation rate of 10 wt% Si, in particular, exhibits a minimum value of only 39.21 μmol O_2_ min^−1^ g^−1^ and 29.96 μmol O_2_ min^−1^ g^−1^, respectively, while YSZ-doped samples maintain significantly higher rates of 89.54 μmol O_2_ min^−1^ g^−1^ and 96.84 μmol O_2_ min^−1^ g^−1^. Doping with a low Si content of 1 wt% results in an increase in the oxidation rate to 100.12 μmol O_2_ min^−1^ g^−1^, while the reduction rate remains high at 123.32 μmol O_2_ min^−1^ g^−1^, which is comparable to that of the undoped sample. When the mass ratio reaches 2 wt%, the reduction rate declines to 109.375 μmol O_2_ min^−1^ g^−1^, while the oxidation rate continues to increase and reaches its maximum level of 105.18 μmol O_2_ min^−1^ g^−1^, which is comparable to that of YSZ at 5 wt%.

The incorporation of dopants also influences the initial temperature of the redox reaction, as demonstrated in Table 1. The onset temperatures of both the reduction and oxidation have an increase with doping YSZ and SiO_2_. The onset temperatures for the reduction and oxidation reactions exhibit an average increase of 12 °C and 6.5 °C, respectively, upon YSZ doping, resulting in a slight increase in the thermal hysteresis except for the case of 10 wt% YSZ where it remains constant. The SiO_2_ doping also exhibits a similar effect, with an increase in the reduction onset temperature of approximately 7.85 °C compared to pure Mn-Fe granules. Particularly, it is noteworthy that there is a significant elevation in the oxidation onset temperature by about 45.7 °C upon SiO_2_ doping, leading to a decrease in hysteresis values and consequently enhancing thermal efficiency. It is also found that the overall reaction enthalpy of the MF group exhibits a lower value than the theoretical value 202 J/g. The reported reaction enthalpy values always vary among the existing research studies, which can be ascribed to the variations in synthesis routes and experimental conditions. Overall, the trend of enthalpy values observed in this study demonstrates a clear decreasing trend with an increasing doping ratio. Therefore, it is crucial to carefully select appropriate doping ratios to maintain an optimal energy storage density.

### 2.2. Redox Performance in Packed-Bed Reactor

**Figure 4:**
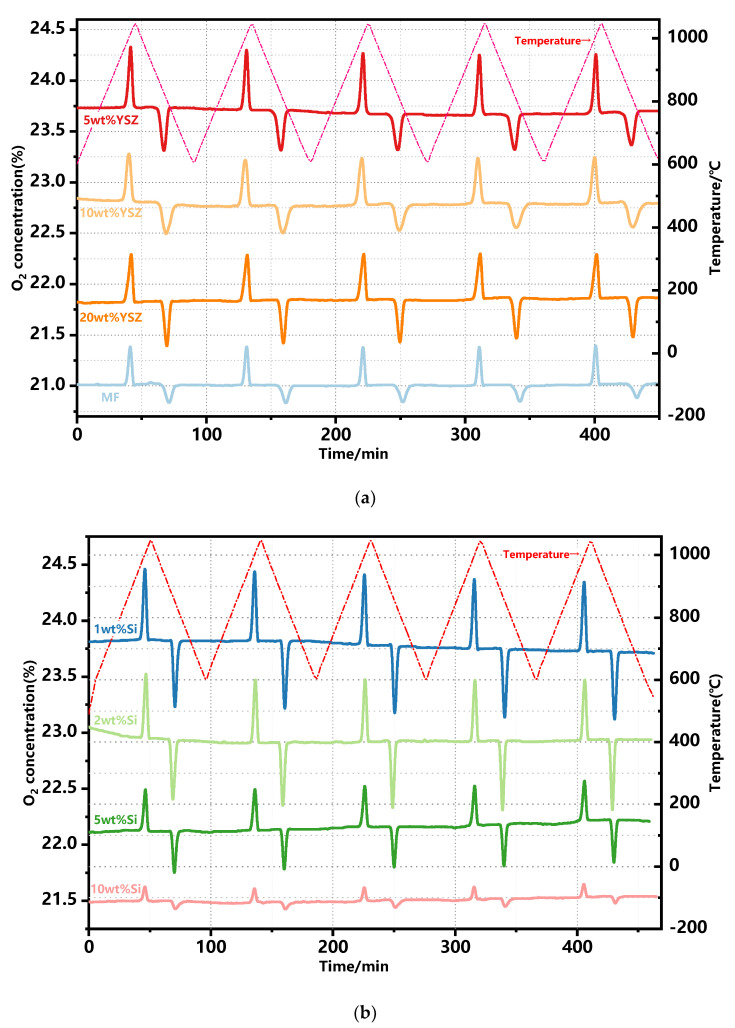
O_2_ concentration profiles of the doped granules over 5 consecutive redox cycles. (**a**) YSZ−doped and MF granules; (**b**) Si−doped granules.

The redox performance of approximately 2 g doped Mn-Fe granules was further assessed in a laboratory-scale packed-bed reactor in the temperature range of 600–1050 °C with a ramp rate of 10 °C min^−1^ and a constant air flow rate of 1 NL·min^−1^. The profiles of the evolution of the outlet O_2_ concentration are continuously monitored throughout the redox reactions, as depicted in Figure 4. Evidently, the oxygen concentration profiles consistently maintain a uniform shape throughout the redox reaction process across all assays, displaying distinct peaks of oxygen release and uptake in each cycle. This observation highlights a remarkable level of repeatability between cycles for the doped Mn-Fe granules, albeit with a slight drift observed during the non-reactive stage.

**Figure 5:**
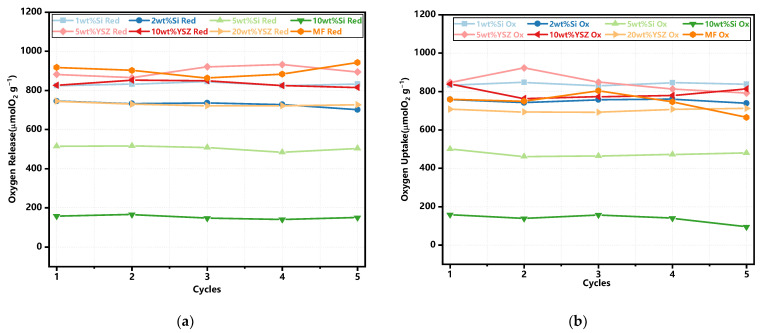
The trends of oxygen release and uptake in the redox reactions. (**a**) The reduction reaction; (**b**) the oxidation reaction.

The trends of oxygen release and uptake in each cycle are illustrated in Figure 5. The thermochemical energy storage performance of the doped granules is also concluded in Table 2. In the case of oxygen release, the pure MF granules undoubtedly exhibit the highest average O_2_ release capability, as depicted in Table 2, which is the same to the TG test results (see Figure 3c). The addition of Si dopant fails to contribute to the O_2_ release process. The Si-doped granules exhibited lower theoretical release values of 79.86% and 70.66% for 1 wt% Si and 2 wt% Si, respectively. When the doping ratio is increased to 5 wt% and 10 wt%, the average O_2_ release values are decreased to 50.57% and 16.14%, respectively. The reason for the decreased amounts of released O_2_ is mainly due to the formation of some kind of manganese–silicon compound, and the deceased reaction process is also consistent with the TG test results (see Figure 3c). The YSZ-doped granules, however, even exhibit a higher theoretical released value of 86.5–90% when compared with the pure and Si-doped granules. It is mainly because YSZ doping has brought about the enhanced concentration of lattice oxygen in the granule, which results in the improved O_2_ storage capacity [37]. Subsequently, the O_2_ release in the reduction reaction process is thus increased. In terms of the average oxygen uptake, all groups with doped granules exhibit a superior performance compared to pure Mn-Fe granules, except for the 5 wt% Si and 10 wt% Si groups, which is also similar to the TG test results. The appropriate levels of doping significantly enhance the oxidation reaction.

**Figure 6:**
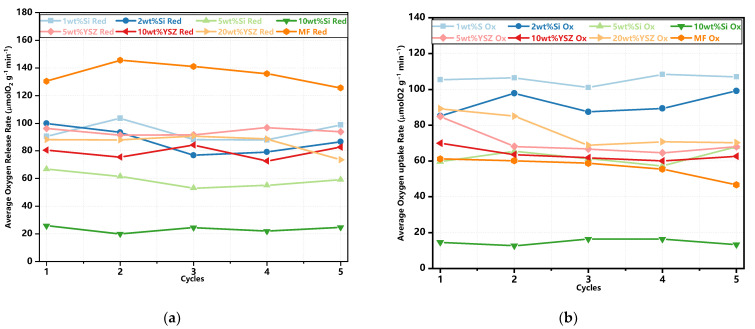
The average oxygen release and uptake rate in the redox cyclic tests. (**a**) The reduction reaction; (**b**) the oxidation reaction.

The average rates of oxygen release and uptake are also illustrated in Figure 6. It is found that the undoped granules exhibit a consistently high average oxygen release rate. Additionally, all doped samples, except for 5 wt% Si and 10 wt% Si, have no significant difference in this rate. When considering the average oxygen uptake rate, all doped granules, with the exception of 10 wt% Si, present a significant enhancement when compared with pure Mn-Fe granules. In particular, 1 wt% Si and 2 wt% Si still possess excellent oxygen uptake rates of 107.1 μmol O_2_ min^−1^ g^−1^ and 99.1 μmol O_2_ min^−1^ g^−1^ after five redox cycles, respectively. Meanwhile, the remaining doped groups maintain similar rates ranging from 62 to 70.2 μmol O_2_ min^−1^ g^−1^. 

### 2.3. Long-Term Redox Cycles’ Performance Analysis

In order to study the cyclic stability and evolution of the micro- and macro-structure of granules, the 120 redox reactions were carried out in a muffle furnace under the same temperature procedure used in the reactor tube, and the granules were sampled for further analysis after every 30 cycled tests. Additionally, only group MF, 1 wt% Si, 2 wt% Si, and 10 wt% YSZ were selected for further experimentation based on the consideration of the better redox performance and energy storage density shown in Section 3.2. After the 120th cycle, the XRD, SEM, and MIP characterizations were conducted to investigate the phase transformation and microstructural evolution of the cycled granules. Meanwhile the crushing strength of the granules was tested before and after the cycles.

**Figure 7:**
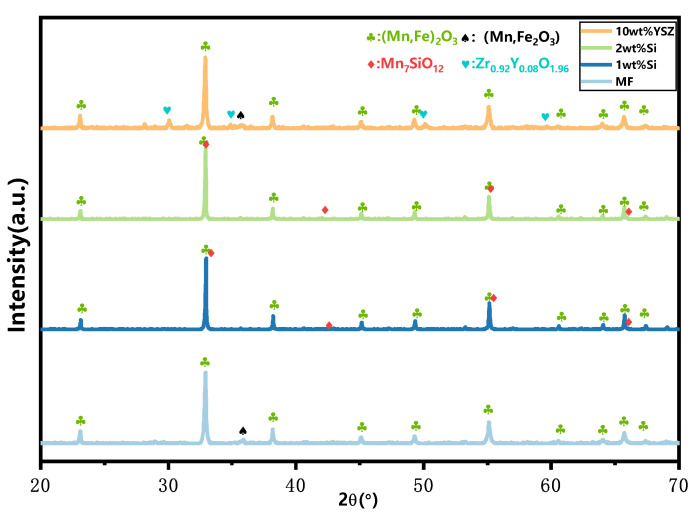
XRD patterns of granules after 120 cyclic tests.

**Figure 8:**
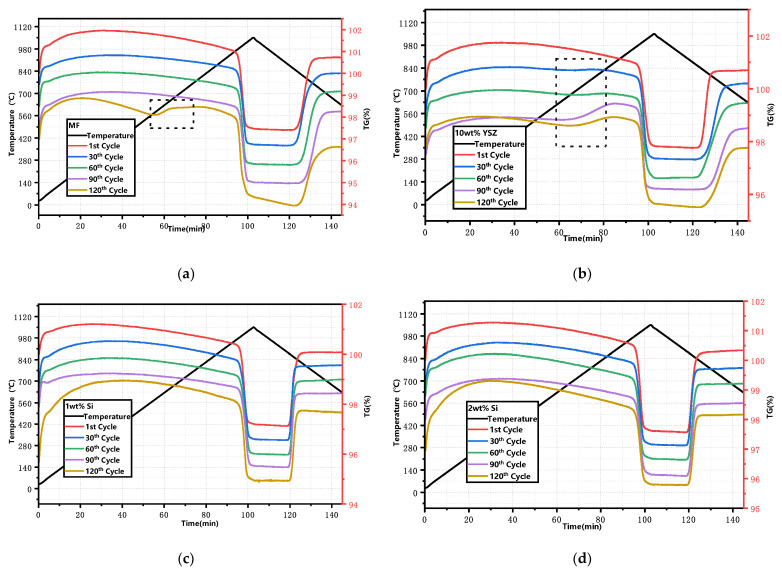
TG curves of the granules after every 30 cyclic tests. (**a**) MF; (**b**) 10 wt% YSZ; (**c**) 1 wt% Si; (**d**) 2 wt% Si (dashed black square: mass gain).

The XRD patterns of the granules after 120 cyclic tests are presented in Figure 7. All Si-doped granules exhibit the presence of phase Mn_2_O_3_ (ICDD; PDF-2; #41-1442) as well as a minor phase, braunite Mn_7_SiO_12_, which is consistent with the observations before the cycle process. However, in the MF and 10 wt% YSZ groups, besides Mn_2_O_3_ (ICDD; PDF-2; #41-1442), there is also evidence of the appearance of a Mn_3_O_4_ phase (ICCD; PDF-2; #01-089-4837), indicating incomplete conversion during the re-oxidation reaction. The thermogravimetric (TG) curves of the sampled granules after every 30 cyclic tests are depicted in Figure 8. These curves have been adjusted for clarity while preserving their original significance. In Figure 8a,b, a slight mass gain (within the range indicated by the dashed black square) is observed in the initial heating program at approximately 580 °C and 650 °C for MF and 10 wt% YSZ, before the initiation of the reduction reaction, respectively. The emergence of this phenomenon is observed for 10 wt% YSZ after 30 cyclic tests, whereas it only manifests for MF after the 120th cycle. The long-term redox cycles appear to have a minimal impact on the reduction reaction of all groups, as evidenced by the nearly vertical weight loss profile observed over 120 cycles. In terms of re-oxidation evolution, both the MF and 10 wt% YSZ groups display a sluggish trend, while the Si-doped group consistently exhibits a stable vertical change in mass gain throughout the 120 cycles. 

**Figure 9:**
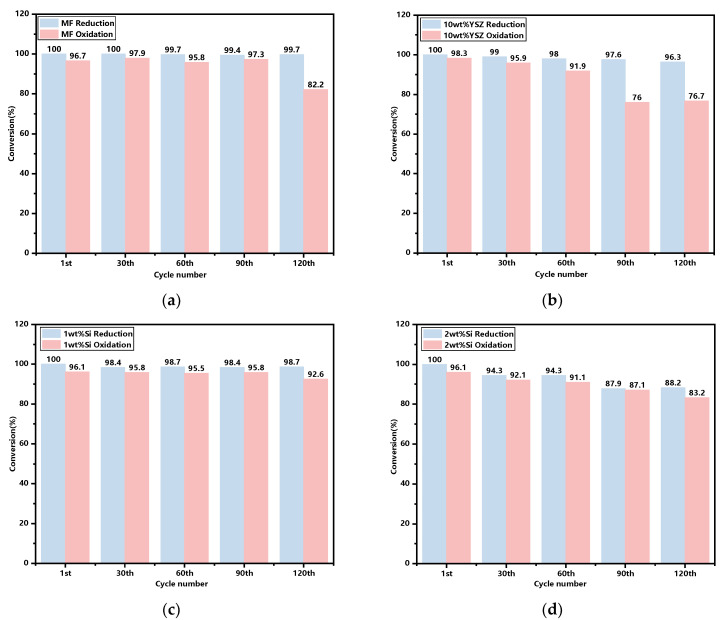
The reduction and oxidation reaction conversions of the granules after every 30 cyclic tests. (**a**) MF; (**b**) 10 wt% YSZ; (**c**) 1 wt% Si; (**d**) 2 wt% Si.

The molar conversions of the sampled granules after every 30 cyclic tests, with the initial cycle serving as a reference, are illustrated in Figure 9. It is found that the reactivities in the reduction reaction are basically consistent for both the doped and undoped groups, with the exception of a slight decrease to 88.2% for the 2 wt% Si after 120 cyclic tests. In terms of the oxidation reaction process, the reactivity of all groups generally decreases over the cycles. However, the Si-doped groups exhibit a more gradual decline trend when compared with both the 10 wt% YSZ and MF groups, which experience rapid declines starting from the 90th and 120th cycle. After 120 cycles, the oxidation reactivities of the Si-doped groups remained at 92.6% and 83.2% for 1 wt% and 2 wt%, respectively, surpassing those of the undoped group (82.2%) and the 10 wt% YSZ group (76.7%).

**Figure 10:**
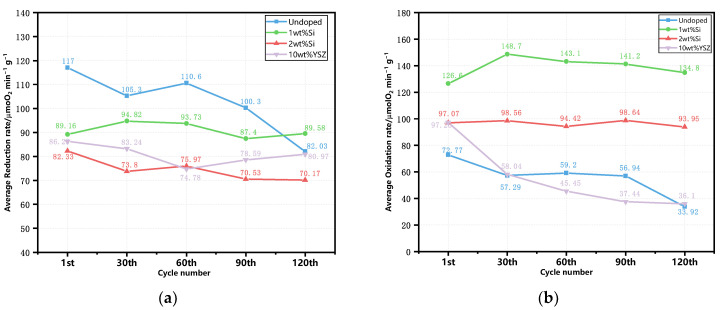
The evolution of average reaction rate of the granules in the cyclic tests. (**a**) The reduction reaction; (**b**) the oxidation reaction.

The evolution of the average reaction rate (μmol O_2_ min^−1^ g^−1^) for reduction and oxidation is illustrated in Figure 10. The undoped granules present the highest average reduction rate, reaching 117 μmol O_2_ min^−1^ g^−1^ among the other doped groups at the beginning of the 120 cycles. However, there is a noticeable decreasing trend with the cyclic tests. In contrast, the doped granules exhibit a lower but stable reduction reaction rate throughout the cycles. In particular, the 1 wt% Si still exhibits a rate of 89.58 μmol O_2_ min^−1^ g^−1^ after 120 cycles, which is comparable to its initial reaction rate of 89.16 μmol O_2_ min^−1^ g^−1^. As for the oxidation process, it is found that doping can enhance the initial average oxidation rate, which is also demonstrated in Section 2.1. But after 30 cycles, both undoped and 10 wt% YSZ groups experience a rapid decline in their reaction rates, which persists until the end of the experiment. In contrast, the Si-doped group maintains its reaction reactivity without any significant decline. Notably, the 1 wt% Si group shows a relatively high and stable rate throughout the 120 cycles, maintaining a value of 134.8 μmol O_2_ min^−1^ g^−1^, which is 3.7 times higher than that of the undoped group. The oxidation reaction rate of the 2 wt% Si group still remains at 93.95 μmolO_2_ min^−1^ g^−1^, second only to that of the 1 wt% Si group and representing a value that is 2.6 times higher than that of the undoped group, despite having the lowest average reduction rate (70.17 μmolO_2_ min^−1^ g^−1^).

**Figure 11:**
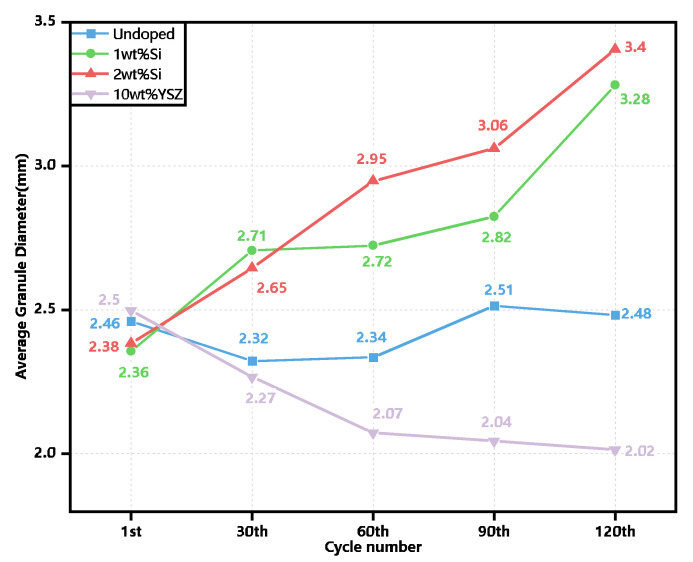
The evolution of granule sizes from the 1st cycle to the 120th cycle.

In addition to assessing the redox performance, the evolution of the granule size for each group was evaluated after every 30 cycles using ImageJ software, employing an analysis of more than 20 granules, as depicted in Figure 11. The initial granule sizes of all groups tend to be similar, ranging from approximately 2.36–2.5 mm prior to the cyclic process. However, noticeable variations in size are observed among the groups after the completion of the cycles. The granule size of the Si-doped groups exhibited a nearly linear increase with the number of cycles, reaching a maximum value of 3.4 mm for 2 wt% Si and the second highest value of 3.28 mm for 1 wt% Si at the end of the cycles, which is nearly 40% and 38.9% larger than its initial state. The increase in size of the Si-doped groups is significantly more pronounced compared to the relatively inconspicuous changes observed in the granule sizes of the undoped group. The 10 wt% YSZ group exhibited an anomalous phenomenon characterized by a contraction in the size of the granules. During the initial 60 cycles, significant and discernible alterations in the granule sizes were observed in both the 10 wt% YSZ and 2 wt% Si groups, with one group exhibiting contraction while the other demonstrated expansion. Subsequently, the contraction rate of 10 wt% YSZ gradually decelerated and eventually stabilized at 2.02 mm, indicating a reduction of nearly 20% compared to its initial state. 

**Figure 12:**
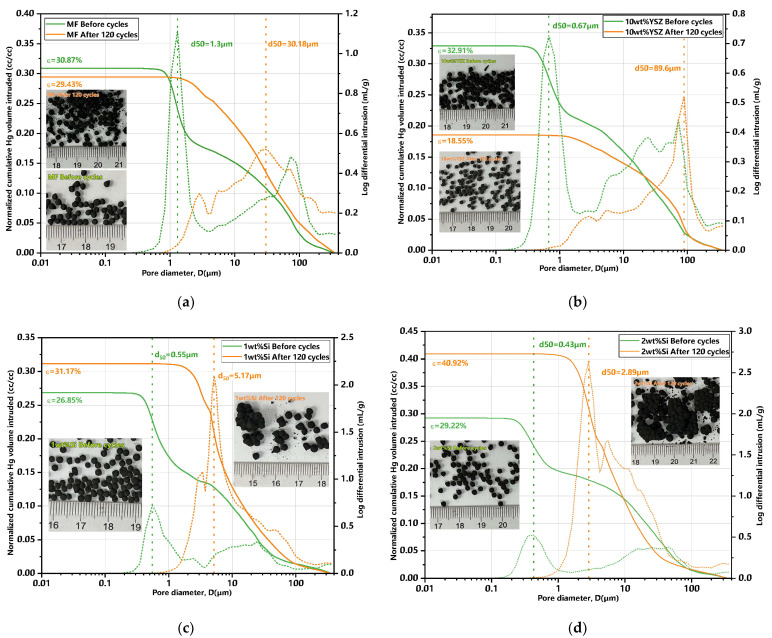
MIP curves of granules for pre- and post-120 cycles and their corresponding granules images. (**a**) MF granules; (**b**) 10 wt% YSZ granules; (**c**) 1 wt% Si granules; (**d**) 2 wt% Si granules (green dotted line: mean pore size before cycles; yellow dotted line: mean pore size after cycles).

Furthermore, MIP characterizations were conducted to investigate the porosity changes of the granules both pre- and post-120 cycles. The findings are summarized in Table 3, accompanied by their corresponding differential and normalized cumulative Hg porosimetry curves (in cc/cc, enabling the calculation of porosity), as well as the associated granule images presented in Figure 12. The initial total porosity is approximately 30% for all groups, except for the 1 wt% Si group which exhibits a slightly lower value of 26.85% compared to the other groups. Also, the initial mean pore size of the doped granules measures less than 1 μm (green dotted line), while the undoped granules have a mean pore size of 1.31 μm (yellow dotted line). The porosity of the Si-doped groups exhibited an approximate increase of 16.1% and 40% for 1 wt% Si and 2 wt% Si, respectively, compared to their initial porosity after 120 cycles. In contrast, the undoped group only showed a negligible increase in porosity. The porosity in the 10 wt% YSZ group exhibited a significant decrease, dropping from 32.91% to 18.5%. The overall porosity trend presents a high degree of consistency with the evolution of granule size. 

The mechanical strength of the granules was also assessed before and after 120 cycles, as presented in Table 3. The initial average crushing strength of all of the groups is approximately ~0.36 N, surpassing the previously reported values of 0.166 N (Bielsa et al. [32]). Notably, the 2 wt% Si group exhibited the highest value of 0.92 N. As previously discussed, the macro-structure of the granules undergoes significant changes in terms of expansion or contraction after the cycles, resulting in considerable variations in their corresponding crushing strength. The crushing strength of all of the groups exhibited a decrease, except for the 10 wt% YSZ group which demonstrated an increase to 2.44 N after the cycles. The crushing strength of the 2 wt% Si and 1 wt% Si groups decreased to 0.48 N and 0.28 N, respectively, surpassing the value of the undoped group (0.11 N), indicating that the Si-doped groups lead to a significant improvement in mechanical enhancement after long-term cycles.

**Figure 13:**
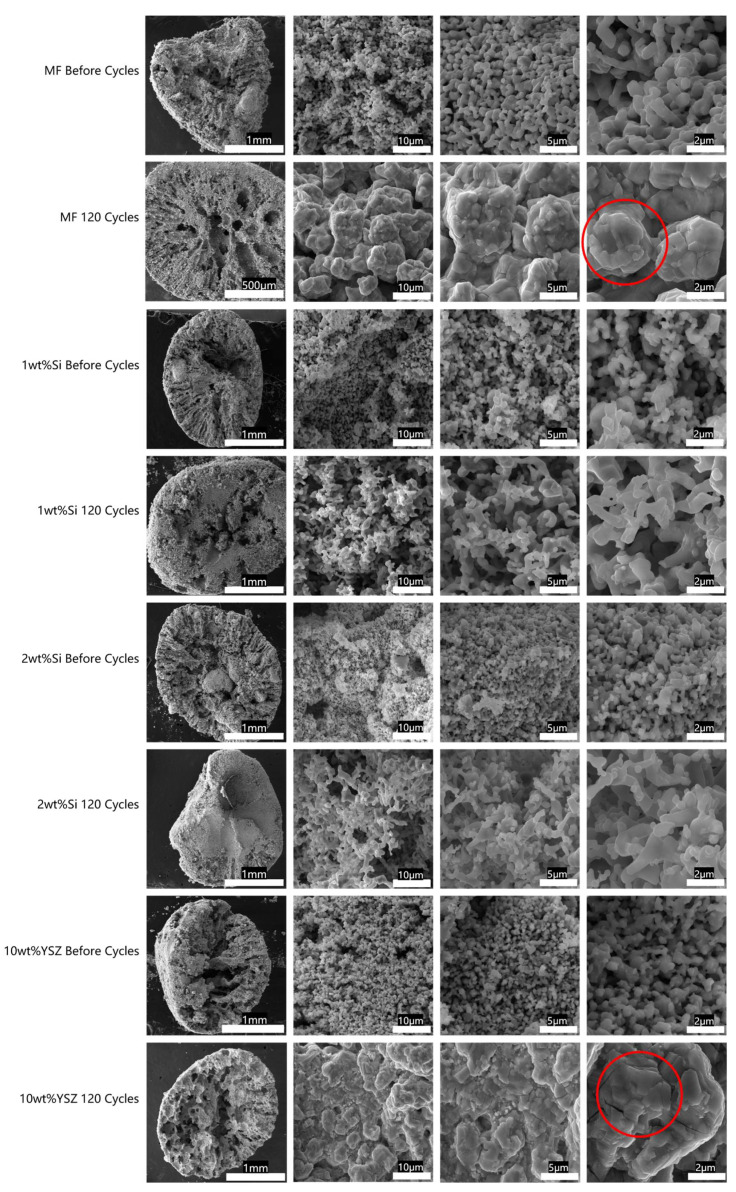
The SEM images of cross-sectional cuts of granules before and after 120 cycles, accompanied by consecutive magnification (red circle: sintering area).

In order to investigate the microstructural evolution of the granules before and after 120 cycles, we also conducted SEM characterizations. The SEM images of cross-sectional cuts of granules are depicted in Figure 13 in consecutive magnifications. 

From the SEM images, it is evident that the granulation process results in the dissolution of organic solvents in water, leading to the formation of channels extending from the surface to the interior and exhibiting grooved patterns at cross-sections. Consequently, the presence of these channels significantly facilitates the ingress of oxygen from the air. However, after undergoing consecutive cycles, the internal structure underwent significant changes under various stresses. The channels in Si-doped granules were clearly eliminated due to expansion, while the internally predominant structure of doped YSZ particles exhibited an interconnected porous architecture despite experiencing significant volume shrinkage during the cyclic process. By further magnifying the local area of the cross-section, it is possible to observe that the grains grow larger than its initial size in all granules. In contrast to the MF and 10 wt% YSZ samples where the grain morphology appears coarse, sharp, and angular due to sintering (the area signed by the red circle), the Si-doped granules, particularly those containing 1 wt% Si, exhibit a porous coral-like structure which indicated a pronounced anti-sintering effect. In general, the sintering process can be classified into liquid-phase sintering and solid-state sintering, with a focus on the latter in this study. The predominant phenomenon observed during solid state sintering is the simultaneous occurrence of densification and grain growth (coarsening), both of which rely on mass transport along the grain boundaries [40]. In fact, the chemical modification of grain boundaries through the introduction of doping cations has been proven to be an effective strategy. SiO_2_, traditionally used as a dopant, aids in preventing sintering. Previously, Bielsa et al. [32] investigated the lower doping ratio of Si cations on Mn oxides and proposed that the sintering rate in Mn_2_O_3_ is governed by ion grain boundary diffusivity, which can be hindered by the segregation of Si^4+^ dopants at the grain boundaries. Additionally, due to its smaller size and higher valence, Si^4+^ dopants can also enhance re-oxidation kinetics and mitigate sintering effects. Recently, Huang et al. [41] investigated the impact of incorporating a surface modifier MnSiO_3_ into (Mn_0_._8_Fe_0_._2_)_2_O_3_ on its anti-sintering properties and the nanoscale MnSiO_3_ particles were effectively immobilized on the surface of (Mn_0_._8_Fe_0_._2_)_2_O_3_, thereby impeding crystal growth and ensuring stability over 1000 cycles.

## 3. Materials and Methods

### 3.1. Granules Synthesis

**Figure 14:**
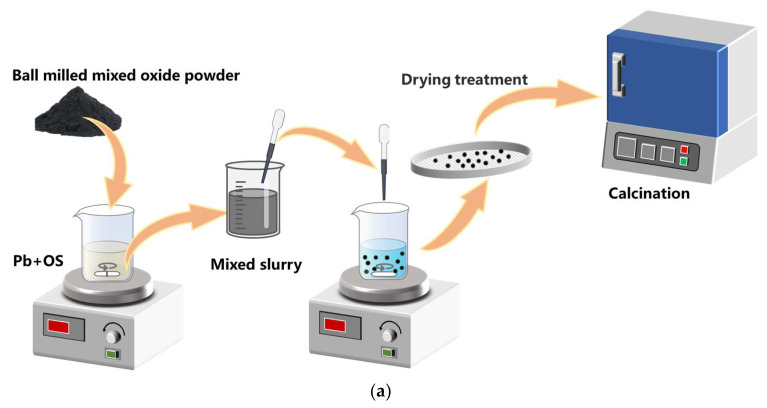

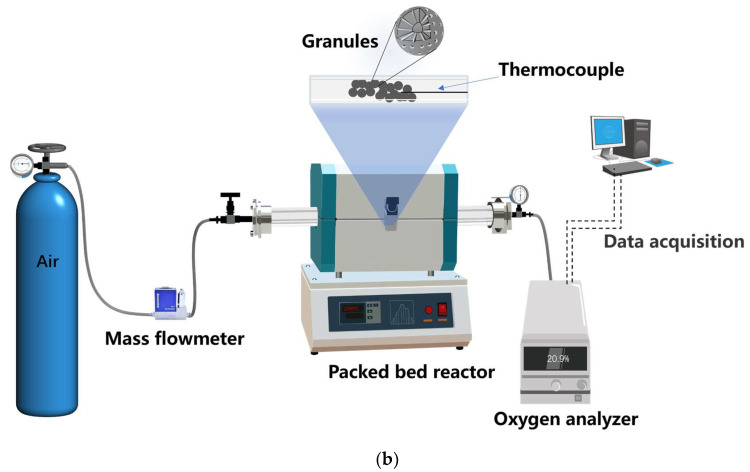
Schematic diagram of the preparation process of Mn-Fe granules and the cyclic tests in a packed reactor. (**a**) The granulation process; (**b**) the cyclic test process.

The preparation process of Mn-Fe oxide granules is shown in Figure 14. The process of granulation primarily comprises three stages: synthesis of Mn-Fe oxides, preparation of granules, and calcination of the granules. 

Synthesis of Mn-Fe oxides. The metal oxides were synthesized using a modified Pechini method as described by Sunde et al. [42] and Jana et al. [43]. The metal precursors, consisting of Mn (NO_3_)_2_ · 4H_2_O (98%, Macklin, Shanghai, China) and Fe (NO_3_)_3_ · 9 H_2_O (>98%, S Macklin), were introduced into an aqueous solution of citric acid (CA, ≥99.5%, Macklin, China) with a molar ratio of Me: CA as 1:5 under continuous stirring for 3 h at a temperature of 70 °C. Subsequently, in order to facilitate the polymerization process, ethylene glycol (EG, ≥99.5%, Macklin, China) was introduced at a molar ratio of CA:EG = 3:2 and the solution was subjected to continuous stirring at 90 °C for 2 h. The gel was subsequently subjected to drying at a temperature of 200 °C for a duration of 3 h, followed by air calcination at 450 °C for a period of 4 h. The calcined gel was finely ground into powder and subsequently subjected to further static air calcination at 700 °C for 4 h with a heating/cooling rate of 2 °C/min, ensuring the formation of the bixbyite crystal structure.

Preparation of granules. The granulation process in this work is implemented using a novel, feasible, and scalable technique known as the “drop technique”, which has been modified by Gigantino et al. [30] and Bielsa et al. [31]. The granules are doped with SiO_2_ (99.9%, Aladdin, Wuhan, China) and 8 mol% Y_2_O_3_-stabilized ZrO_2_ (YSZ, Aladdin, China), respectively. The Mn-Fe oxides powder, mixed with the dopants of SiO_2_ (at 1 wt%, 2 wt%, 5 wt%, and 10 wt%) and YSZ (at 5 wt%, 10 wt%, and 20 wt%), was homogenized in a planetary ball mill operating at a speed of 600 rpm for 30 min, resulting in the powder with an average particle size of 0.05 μm. In this work, MF stands for pure Mn-Fe oxide granules and the abbreviation for doped granules is represented by the doping mass ratio combined with the dopant, such as 1 wt% Si or 5 wt% YSZ, etc.

The polymeric binder (PB) and organic solvent (OS) were selected from ethyl cellulose (viscosity of 18–22 cP, 5% in toluene/ethanol 80:20 vol%, Macklin, China) and 1-methyl-2-pyrrolidinone (>99% purity, Macklin, China), respectively. The mixed metal oxides powder (MO) was added to a solution comprising a polymeric binder (PB) dissolved in an organic solvent (OS) with a mass ratio of MO:PB:OS = 3:1:9. The slurry was preheated to 45 °C in order to reduce its viscosity before being dispensed through a syringe needle with a tip diameter of 2 mm into a precipitating bath composed of deionized water and the surfactant Tween 80 (Macklin, China) (0.2 mL/L), which effectively lowers the surface tension of water. In practical experiments, we observed that the length of the metal needle plays a critical role in slurry flow dynamics. In particular, the utilization of longer needles has been observed to induce a rapid decrease in slurry temperature, leading to an elevation in viscosity and subsequent obstruction that hinders slurry flow. Moreover, achieving precise control over the rate of dropping by manipulating the syringe plunger becomes challenging, particularly when encountering needle blockage. Therefore, we selected a disposable dropper made of polyethylene plastic with a 2 mm tip diameter instead of employing a syringe equipped with a metal needle, as it provides enhanced ease and intuitive control over the drop rate. Moreover, in cases of blockage, it is more convenient to trim the obstructed portion of the dropper rather than replacing the metal needle. The height between the dropper tip and the precipitation bath is maintained at 3 cm to ensure that the granules possess sufficient kinetic energy to penetrate the bath surface and any deformation upon reaching the surface of the water was prevented. The polymer within the droplets underwent solidification while the solvent diffused into the surrounding bath water. 

Calcination of granules. The granules, which possessed a regular spherical shape and were filtered using a sieve, were dried overnight in ambient air. Subsequently, the granules were subjected to calcination in air at a temperature of 1050 °C for 4 h, employing a heating ramp of 2 °C/min. This thermal treatment aimed to eliminate the organic matter, enhance the granules’ strength, and induce the development of a porous structure within them.

### 3.2. Characterization of Granules

The powder X-ray diffraction (XRD) analyses were conducted using a Bruker D8 Advance diffractometer with Cu Kα radiation (λ = 1.5406 Å), covering a diffraction angle (2θ) range of 10–80° with a step size of 0.02°. The crystal phases were identified using Jade 6.0 software, with reference to the ICDD PDF-2 database. The microstructure and morphology of the granules were examined before and after redox cycles using scanning electron microscopy (SEM) with a TESCAN MIRA microscope equipped with a tungsten source at an accelerating voltage of 15 kV. X-ray Photoelectron Spectroscopy (XPS) of the granules was recorded using nonmonochromatic Al Kα radiation (1486.8 eV) using a Thermo Fisher Nexsa X-ray Photoelectron Spectrometer (Thermo Fisher Scientific, Waltham, MA, USA). All binding energies were referred to the C 1s peak at 284.6 eV to compensate for the effect of surface charge. The core-level spectra were curve-fitted into their possible components using Gaussian–Lorentzian peaks after subtracting a Shirley background with the Avantage program 5.9. The granules were subjected to pore size characterization using an AutoPore V 9600 mercury intrusion porosimeter from Micromeritics Instrument Corporation (Norcross, GA, USA), with a sample mass of approximately 600 mg, a contact angle of 130°, and a maximum pressure of 61,000 psia. The average diameter of granules was determined by analyzing more than 20 granules using ImageJ software v1.54, a widely used image processing program. The crushing strength of selected granules before and after 120 cycles was also examined using the SENS CMT6000 apparatus. 

### 3.3. Redox Reactivity of Granules

The redox reaction of a selected composition with a Mn/Fe molar ratio of 3:1 can be described as follows [19]:

(1) $$6{\left({\mathrm{Mn}}_{0.75}{\mathrm{Fe}}_{0.25}\right)}_{2}O_{3}⇌\;4{\left({\mathrm{Mn}}_{0.75}{\mathrm{Fe}}_{0.25}\right)}_{3}O_{4}+O_{2}$$

The reaction kinetics and chemical stability of the granules at different doping ratios were investigated using the simultaneous thermal analyzer STA 449 F3 Jupiter (Netzsch, Bayern, Germany). Each sample, consisting of 3–4 granules weighing approximately 10 mg, was placed into 85 μL open Al_2_O_3_ crucibles (Netzsch). These samples underwent charging and discharging cycles under a synthetic air stream flowing at a rate of 100 mL/min. The molar conversion for reduction and oxidation is defined as

(2) $$X_{\mathrm{Red}}=\frac{6\cdot {∆n}_{O_{2}}M_{{\left({\mathrm{Mn}}_{0.75}{\mathrm{Fe}}_{0.25}\right)}_{2}O_{3}}}{m_{{\left({\mathrm{Mn}}_{0.75}{\mathrm{Fe}}_{0.25}\right)}_{2}O_{3}+\;\mathrm{dopant},\;\mathrm{tot}}\cdot w_{{\left({\mathrm{Mn}}_{0.75}{\mathrm{Fe}}_{0.25}\right)}_{2}O_{3}}}$$

(3) $$X_{\mathrm{Ox}}=\frac{4\cdot {∆n}_{O_{2}}M_{{\left({\mathrm{Mn}}_{0.75}{\mathrm{Fe}}_{0.25}\right)}_{3}O_{4}}}{m_{{\left({\mathrm{Mn}}_{0.75}{\mathrm{Fe}}_{0.25}\right)}_{3}O_{4}+\;\mathrm{dopant},\;\mathrm{tot}}\cdot w_{{\left({\mathrm{Mn}}_{0.75}{\mathrm{Fe}}_{0.25}\right)}_{3}O_{4}}}$$

where the $M_{i}$, $w_{i}$, and $m_{i+\mathrm{dopant},\;\mathrm{tot}}$ represent the molar mass, mass fraction, and total mass, respectively, of species i. ${∆n}_{O_{2}}$ stands for the amount of reacted moles of oxygen which can be calculated by ${∆n}_{O_{2}}={∆m}_{O_{2}}/M_{O_{2}}$. 

As for the cyclic tests in a lab-scale packed-bed reactor, a tube reactor type was adopted. The quartz tube reactor (Φ 13 mm × 1200 mm) was positioned within an electrical furnace, with both ends of the tube hermetically sealed using T-type flanges to facilitate the insertion of a Type-K thermocouple (Φ = 1.5 mm) and efficient gas efflux towards an oxygen analyzer. Approximately 2 g of granules were placed in the middle of the tube reactor. The temperature of the granules was measured by the thermocouple which was inserted at the center of the granules, and the signal was transmitted to a temperature programmer/controller Yudian AI-888 with an accuracy of ±0.01 °C during the experiment. The oxygen concentration downstream of the reactor during the redox cycles was measured using an oxygen analyzer CI-PC926 (Changai, Shanghai, China). The process of the cyclic tests in the packed-bed reactor is depicted in Figure 14b. The molar conversions of granules during the cyclic tests in reactor can also be determined by Equations (2) and (3), and the corresponding ${∆n}_{O_{2}}$, which is determined by integrating the molar flow rate of O_2_ release/uptake during the reduction/oxidation, defined as:

(4) $${∆n}_{O_{2}}=\int_{\mathrm{begin}}^{\mathrm{end}}{\dot{n}}_{O_{2},\mathrm{reacted}}(t)dt$$

(5) $${\dot{n}}_{O_{2},\mathrm{reacted}}=\frac{{\dot{V}}_{\mathrm{air},\mathrm{in}}}{\hat{V}}\cdot \frac{y_{O_{2},\;\mathrm{out}}-y_{O_{2},\;\mathrm{in}}}{{1-y}_{O_{2},\;\mathrm{out}}}$$

where ${\dot{V}}_{\mathrm{air},\mathrm{in}}$, $\hat{V}$, and $y_{i}$ represent the inlet’s overall air flow rate at the inlet, the molar volume of an ideal gas at *T* = 273.15 K and total pressure *p* = 101.325 kPa (Standard Temperature and Pressure = STP), and the oxygen molar fraction, respectively. The average rate of reduction and oxidation (μmolO_2_ min^−1^ g^−1^) can be defined as the amount of oxygen released or taken up during the reaction divided by the corresponding reaction time per unit mass of granules.

## 4. Conclusions

In the TCES application, it is essential for energy storage materials to have adequate mechanical strength and stable reactivity to ensure the long-term operation under the conditions of the high temperature and alternating redox atmosphere. In this study, we synthesized Mn-Fe oxide granules with an average particle size of approximately 2 mm using the drop technique, incorporating SiO_2_ and YSZ at the mass ratios ranging from 1 wt% to 20 wt%. In the TG test, the average reduction rate of the doped granules exhibited a deceleration, while the average oxidation rate experienced a significant enhancement when compared to undoped granules, except for 5 wt% Si and 10 wt% Si. Throughout the five consecutive cycles in the packed-bed reactor experiment, the doped oxides presented stable enhanced reoxidation rates when the doping ratios were in an appropriate range, and decreased reduction rates and energy storage densities. After 120 long-term cyclic tests, the Si-doped granules possessed a better anti-sintering property than that of YSZ-doped granules. Meanwhile, the 1 wt% Si granules exhibited the highest re-oxidation ratio of 92.6%, followed by the second highest ratio of 83.2% for 2 wt% Si. In summary, the incorporation of a small amount (≤1 wt%) of SiO_2_ demonstrates the most optimal TCES performance when considering all factors. However, in the long-term operation under high temperatures, the volume expanding of the Si-doped granules would lead to an increment of pressure drop in the packed-bed reactor, which should be focused. In addition, further dedicated efforts towards enhancing the mechanical strength in the Mn-Fe-Si system are necessary.

## Figures and Tables

**Table 1:** Thermochemical performance of the doped granules based on the TG testing.

Sample	Estimated Mass Change (%)	Experimental Mass Change (%)	*T*_red_/*T*_ox_ (°C)	Δ*T*_Hysteresis_ (°C)	Δ*H*_red_/Δ*H*_ox_ (J/g)
MF	3.37	3.37	983.3/830.3	153	123.4/−144
1 wt%Si	3.33	3.09	990.9/877.1	113.8	90.12/−91.35
2 wt%Si	3.30	2.80	989.5/875.2	114.3	74.95/−103
5 wt%Si	3.20	1.87	992.3/868.3	124	51.22/−67.11
10 wt%Si	3.03	1.27	991.9/883.4	108.5	37.97/−33.9
5 wt%YSZ	3.19	3.21	997.7/840.3	157.4	85.14/−114.2
10 wt%YSZ	3.03	2.96	988.1/835.6	152.5	77.19/−111.5
20 wt%YSZ	2.69	2.64	1000.6/834.5	166.1	65.39/−101.4

**Table 2:** Thermochemical energy storage performance of the granules in packed-bed reactor.

Sample	Average O_2_ Release/Uptake (μmol/g)	Theoretical Release/Uptake (%)	Reactor Average Reduction/Oxidation Duration (min)	TG Reduction/Oxidation Duration (min)
MF	902.67/744.89	85.79/70.79	8.90/7.94	8.45/14.75
1 wt%Si	831.82/838.86	79.86/80.53	8.43/8.22	7.83/9.27
2 wt%Si	728.60/751.16	70.66/72.85	8.59/7.66	8.10/7.99
5 wt%Si	505.46/475.34	50.57/47.55	6.60/9.42	9.06/7.55
10 wt%Si	152.86/137.79	16.14/14.55	9.57/12.11	10.12/10.74
5 wt%YSZ	899.68/845.40	90.01/84.58	10.57/15.79	9.63/9.52
10 wt%YSZ	833.92/793.59	88.06/83.80	8.55/9.26	10.33/9.39
20 wt%YSZ	729.35/702.36	86.65/83.44	6.68/13.25	10.33/8.33

**Table 3:** Analyses of MIP and mechanical strength (main parameters of interest) of fresh and used granules.

Sample	Bulk Density Fresh/Used (g/cm^3^)	Total Porosity Fresh/Used (%)	d_50_ Fresh/Used (μm)	Average Crushing Strength Fresh/Used (N)
MF	0.99/0.98	30.87/29.43	1.3/30.18	0.36/0.11
1 wt%Si	1.12/0.56	26.85/31.17	0.55/5.17	0.39/0.28
2 wt%Si	1.08/0.41	29.22/40.92	0.43/2.89	0.92/0.47
10 wt%YSZ	0.94/1.61	32.91/18.55	0.67/89.6	0.37/2.44

## Data Availability

Data are contained within the article.
